# Does this child just have an atrial septal defect? More potentiality of interventional therapy: A rare case report

**DOI:** 10.1016/j.ijscr.2024.109783

**Published:** 2024-05-22

**Authors:** Yunguo Zhou, Sijia Liu, Jiali Feng, Fang Xu, Junkai Duan, Fei Xu

**Affiliations:** a Jiangxi Provincial Children's Hospital, Nanchang, China; b Nanchang University, Nanchang, China; c JXHC Key Laboratory of Children's Cardiovascular Diseases, Nanchang, China

**Keywords:** Partially anomalous pulmonary venous connection, Vertical vein, Transcatheter closure, Atrial septal defect, Congenital heart disease

## Abstract

**Introduction and importance:**

Partially anomalous pulmonary venous connection (PAPVC) is a rare congenital heart disease, often concomitant with atrial septal defects (ASDs). PAPVC usually tends to be treated by surgery, but the case we report will open up new perspectives for the interventional treatment of PAPVC present with ASD.

**Case presentation:**

We present a case of a 2-year-old 11 kg boy transthoracic echocardiography showed secundum-type ASD. A supracardiac-PAPVC was accidentally detected during cardiac catheterization, and an abnormal pulmonary vein connection was detected with a vertical vein (VV) opening. Ultimately, ASD and VV were both occluded.

**Clinical discussion:**

Surgical therapy of PAPVC is the first line treatment of most centers in the world. However, the main complications after surgical repair of PAPVC raise our concerns, such as pulmonary stenosis, caval vein stenosis and sinus node dysfunction. Therefore, percutaneous closure of PAPVC can be an alternative method. This case of percutaneous interventional closure of ASD and supracardiac PAPVC through a vertical vein in the same surgery was first reported. Patients with ASD tend to have missed diagnoses of PAPVC. We can evaluate it by transesophageal echocardiography (TEE), cardiac magnetic resonance imaging (CMR) and computed tomography (CT).

**Conclusions:**

This case suggests that the effect of interventional therapy is quite reliable. For children with ASD, attention should be paid to the omission of the presence or absence of PAPVC before surgery. During interventional therapy, a guide wire rather than a catheter should be preferred to explore the atrial septum and pulmonary veins to avoid a missed diagnosis of PAPVC.

## 1 Introduction

Partial anomalous pulmonary venous connection (PAPVC) is a congenital heart disease in which some (but not all) pulmonary veins connect to the right atrium or one or more of its venous branches. According to reports, the prevalence of PAPVC is as high as 0.7 % in autopsy series and as high as 10 % in ASD patients [1]. At present, surgical correction is mainly used. However, due to the advantages of minimal trauma, fewer surgical complications, and faster recovery, the number of patients with congenital heart disease treated with interventional catheterization continues to increase. In the past decade, there have been reported of interventional treatment for some left upper pulmonary vein connections and vertical veins [2,3]. We will also applied transcatheter intervention therapy to some relatively rare cases in clinical practice.

Here, we report a special case which preoperative transthoracic echocardiography showed a central type's ASD. First, the PAPVC was accidentally detected when use guide wire to probe the left upper pulmonary vein. Second, we found that the left superior pulmonary vein drained to the left atrium and vertical vein. Finally, VV and ASD were transcutaneously occluded.

This work was reported in line with the SCARE criteria [4].

## 2 Case presentation

**Fig. 1:**
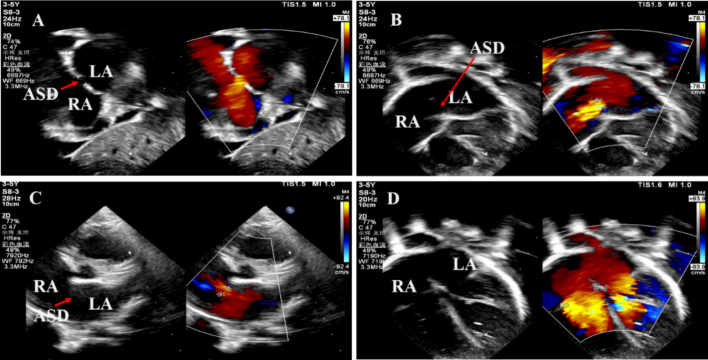
A: A left to right flow can be seen crossed ASD in the subcostal two atrium chamber view by 2D transthoracic echocardiography with color Doppler. B: A left to right flow was shown across ASD in the apical four-chamber view by 2D transthoracic echocardiography with color Doppler. C: A left to right flow was shown across ASD in the parasternal short axis view by 2D transthoracic echocardiography with color Doppler. D: The echocardiography displayed the enlargement of RA and right ventricle. Red arrows indicate the ASD. Notes: LA: left atrium; RA: right atrium; ASD: atrial septal defect. (For interpretation of the references to color in this figure legend, the reader is referred to the web version of this article.)

A two-year-old 11 kg boy was admitted to our medical unit after hearing a persistent systolic cardiac murmur at the second and third intercostal margins of the left sternum, and oxygen saturation measured in indoor air is 100 %. There was no family history of cardiovascular disease and no history of heart disease treatment. The electrocardiogram showed sinus rhythm and right ventricular hypertrophy. The chest radiography showed cardiomegaly and increased pulmonary vascular markings. Transthoracic echocardiography (TTE) showed a secundum-type ASD with 6 mm in diameter (Fig. 1A–C). The diameter of the right atrium and right ventricle is above the normal range (Fig. 1D). According to guidelines for cardiac catheterization and intervention in pediatric cardiac disease: a scientific statement from the American Heart Association [5], patients over two years of age and weight more than 10 kg should receive interventional treatment. Furthermore, the parents strongly requested interventional treatment and signs informed consent.

**Fig. 2:**
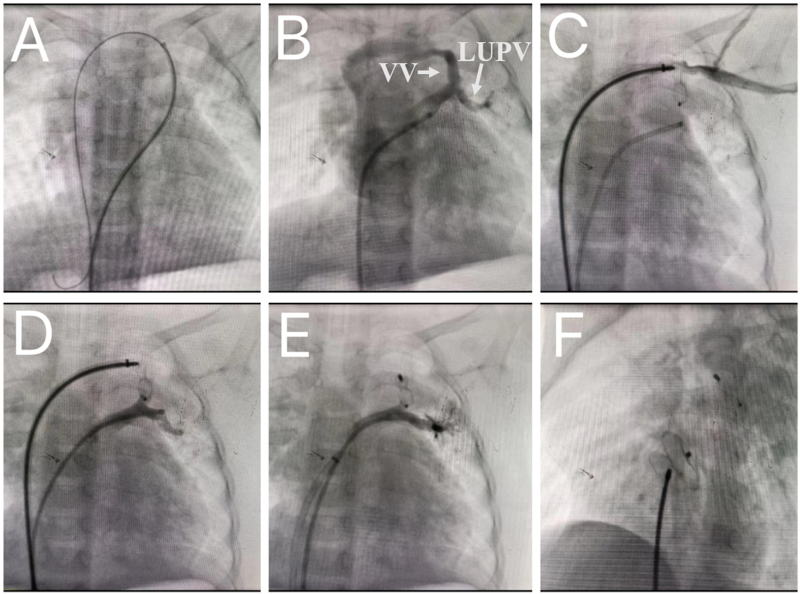
A: The guide wire can pass through the VV, brachiocephalic vein, superior vena cava, and right atrium successively from the left atrium. B: Contrast injection in the LUPV showed that contrast in the LUPV approached the brachiocephalic vein through the VV and flowed into the superior vena cava and the right atrium. C: Left upper pulmonary venography indicates that the LUPV blood returns to the left atrium without obstruction after occlusion. D: Axillary phlebography indicates no obstruction of axillary vein blood flowing back to the left cephalbrachial vein. E: Left upper pulmonary venography indicates that the pulmonary vein flows back to the left atrium. F: Radiography after vertical vein occlusion and atrial septal defect closure. Notes: VV: vertical vein; LUPV: left upper pulmonary vein.

**Fig. 3:**
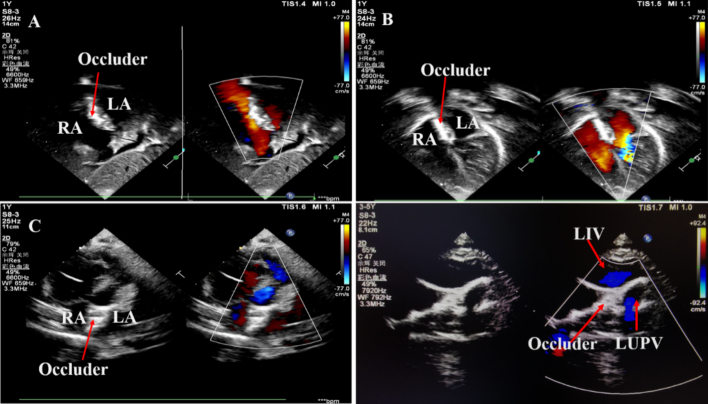
A–C: The occluder is visible in the atrial septum, with good shape and no residual shunt in the three views (subcostal two atrium chamber view, apical four-chamber view, parasternal short axis view) by 2D transthoracic echocardiography with color Doppler. D: The occlusion device is in the vertical vein and there is no obstruction of subclavian artery flow in the suprasternal fossa view by 2D transthoracic echocardiography. Notes: LA: left atrium; RA: right atrium; ASD: atrial septal defect. LIV: left innominate vein; LUPV: left upper pulmonary vein.

Under general anesthesia, 100 IU/kg intravenous heparin was administered and percutaneous closure of ASD was performed. We selected a 6-Fr MPA2 catheter and inserted it into the right femoral vein. After conventional cardiac catheterization, the average pressure in the right and left atria was 6 and 9 mmHg, respectively. Blood samples were collected from the inferior vena cava, superior vena cava, pulmonary vein, and pulmonary artery for blood gas analysis. The oxygen saturation values were 0.79, 0.85, 0.99, and 0.87, respectively. Therefore, Qp/Qs was calculated as 1.75:1. Accidentally, when MPA2 was sent into the left atrium through the ASD, an abnormal channel was discovered while probing the left superior pulmonary vein with a 0.035-in × 260-cm J-tipped Radifocus® Guide wire (Terumo Corp, Tokyo, Japan), which could extend to the veins of the superior cavity, the right atrium, and the inferior vena cava (Fig. 2A). Given the possibility of a partially anomalous pulmonary venous connection, angiography was performed in the left superior pulmonary vein. It showed that part of the blood flow in the left superior pulmonary vein approached the brachiocephalic vein through the VV (Fig. 2B). After entering the right atrium through the superior vena cava, a partial supracardiac connection to the left superior pulmonary vein was diagnosed. After puncturing the left femoral vein, we used a guide wire and an MPA2 to reach the left atrium from the inferior vena cava via the right atrium, the superior vena cava, the brachiocephalic vein, the VV and the left superior pulmonary vein. We fix the distal end of MPA2 in the left atrium, exit the soft guide wire, withdraw MPA2 after entering the hard guide wire, and send the prepared 7F long sheath to the left atrium along the hard guide wire. The inner diameter of the vertical vein is approximately 3.5 mm and the length is approximately 8 mm. The 6/8 mm patent ductus arteriosus occluder (Shape Memory Alloy Corp, shanghai, China) was selected to block the VV. Before releasing the occluder, axillary venous angiography showed no obstruction in blood flow from the left subclavian vein to the brachiocephalic vein (Fig. 2C). Meanwhile, the left upper pulmonary vein angiography showed that the left upper pulmonary vein returned smoothly to the left atrium (Fig. 2D). After the occluder was released, an angiography was performed again to confirm that no obstruction was found (Fig. 2E). The ASD was occluded with a 12 mm waist diameter occluder. Finally, ASD and VV were closed with interventional therapy (Fig. 2F). Postoperative fluoroscopy and ultrasound examination showed that the occluders were in good position, without residual shunt or mitral regurgitation, and the blood flow velocity of the vena cava and pulmonary vein was normal. On the second day after the operation, enteric coated tablets with aspirin (3 mg/kg·d) were taken orally for anticoagulation and no abnormalities were found in any echocardiography, electrocardiogram, or chest radiograph. All above three examinations were normal at 18-month follow-up (Fig. 3).

## 3 Discussion

From an embryological perspective, the lungs originate from the foregut. In the early stages of fetal development, the pulmonary and systemic venous circulation is directly connected. The persistence of venous drainage of the lung into the systemic cardinal and umbilical vitelline systems results in a variety of anomalies of pulmonary venous return [6]. There are three different types of PAPVC, and the location of the drainage determines whether it is supracardiac, cardiac, or intracardiac [7]. The supracardiac type drains to the superior vena cava (SVC), the intracardiac type drains directly to the right atrium or innominate vein, and the subcardiac type, the rarest variant, drains to the inferior vena cava or portal vein [8]. There are several surgical techniques for the repair of PAPVC [9]. Single-patch, double-patch, and Warden procedures, also known as cavalry division, are commonly used. Surgical therapy of PAPVC is the first line treatment in most centers around the world [10]. However, the main complications after surgical repair of PAPVC raise our concerns, such as pulmonary vein stenosis, vena cava stenosis, and sinus node dysfunction. Due to these complications, some patients may require a second surgery to expand the vena cava or implant a pacemaker to treat sinus node dysfunction [11,12]. Therefore, if percutaneous closure of PAPVC can be used, it is an alternative treatment method.

Atrial septal defect accounts for 7 % of congenital heart disease and can affect growth and development, even leading to stroke [5]. In addition, there is also a relationship between ASD and the development of atrial fibrillation. Electromechanical delay may lead to an increased trend of atrial fibrillation. Compared to healthy individuals, electromechanical delay in patients with ASD can be reduced after receiving occlusion therapy, thereby reducing the occurrence of atrial fibrillation [13]. Atrial septal defect can coexist with PAPVC. The clinical manifestations are similar and can be asymptomatic. Both cardiac color Doppler ultrasound images show enlargement and reconstruction of the right heart system caused by left to right shunting. Therefore, distinguishing between ASD and PAPVC in clinical practice is challenging. Fortunately, surgical corrections of ASD and PAPVC have good outcomes with a low complication rate and typically lead to an immediate resolution of symptoms. But infants with Scimitar syndrome have a poorer outcome due to complex anatomy and the presence of other co-existing cardiac anomalies [14].

To our knowledge, our patient was the first reported case of percutaneous interventional closure of both ASD and VV in the same surgery, thereby curing ASD and epicardial PAPVC. Patients with ASD tend to been missed diagnoses of PAPVC. The missed diagnosis rate of PAPVC is unclear, but it is more likely to be missed when combined with ASD [15]. It is estimated that 10 % to 15 % of patients with secondum-type ASD and up to 85 % of patients with sinus venous-type ASD have associated PAPVC [16]. The leading causes of missed diagnosis of PAPVC are undefined cardiac structural deformities prior to operation and the lack of careful exploration during the operation. These misdiagnosed patients usually require secondary corrective surgery or transcatheter intervention treatment [17,18].

Generally, TTE is the preferred method for diagnosing ectopic pulmonary vein drainage, but due to the distance between the pulmonary vein and conventional ultrasound views, the number of pulmonary vein branches, technology, and operator experience, as well as the size and quality of the sound window, TTE used for diagnosing PAPVC and identifying the site of SVC drainage has a high misdiagnosis and missed diagnosis error rate [19]. We can evaluate it through transesophageal echocardiography (TEE), cardiac magnetic resonance imaging (CMR), and computed tomography (CT), which can reveal all vascular connections in the thorax without any anatomic limitations and are more suitable for identifying PAPVCs [20].

In addition, TEE should fully evaluate the diameter of the right atrium and right ventricle. If the size of the defect does not match, we should consider whether there is an unknown congenital heart disease. During surgery, the loach guide wire can be used to examine the left upper pulmonary vein and improve the detection rate of some abnormal left pulmonary vein connections. We suggest that before and after vertical vein occlusion, attention should be paid to angiography and color Doppler ultrasound to confirm that the occluder did not obstruct the left upper pulmonary vein and subclavian vein. Otherwise, it is necessary to readjust the position of the occluder.

The selection of occluder is determined based on the size and shape of VV [2]. Some scholars suggest oversizing by 30–50 % to the diameter measured at catheterization [21]. The general choice is 1.6 to 1.8 times the inner diameter of the narrowest part, and some different researchers believe that adding 2 mm to the inner diameter of the narrowest part is also feasible [22,23]. To avoid oversizing the device selection, we chose an occluder size of 1.7 times the inner diameter of the narrowest part, and the results are reliable.

## 4 Conclusion

This case demonstrates that the effectiveness of interventional treatment is quite reliable. For children with ASD, attention should be paid to whether PAPVC is missed before surgery. During interventional therapy, guide wires should be preferred over catheters to explore the atrial septum and pulmonary veins, in order to avoid missed diagnosis of PAPVC.

## Informed consent

Written informed consent was obtained from the patient for publication of this case report and accompanying images. A copy of the written consent is available for review by the Editor-in-Chief of this journal on request.

## Ethical approval

Ethical approval for this study was provided by the Ethical Committee of Jiangxi Provincial Children's Hospital, Nanchang, China on 1 March 2022 (JXSETYY-YXKY-20220164).

## Funding

This work was supported by the 10.13039/501100001809National Natural Science Foundation of China (No. 81860065) and the Science and Technology Planning Project of Health Commission of Jiangxi Province, China (No. 202130919).

## Research registration number

Not applicable.

## Guarantor

Yunguo Zhou.

## Credit authorship contribution statement

Yunuo Zhou designed this study. Sijia Liu, Jiali Feng and Fang Xu conducted the artical collection and analysis. Sijia Liu drafted the manuscript which was checked by Junkai Duan and Fei Xu. All authors contributed to the article and approved the submitted version.

## Declaration of competing interest

The authors declare that they have no competing interests.

## Data Availability

All data generated or analysed during this study are included in this published article.
